# Comparative study on contrast enhancement of Magnevist and Magnevist-loaded nanoparticles in pancreatic cancer PDX model monitored by MRI

**DOI:** 10.1186/s12645-020-00061-9

**Published:** 2020-05-14

**Authors:** Kevin Affram, Taylor Smith, Shannon Helsper, Jens T. Rosenberg, Bo Han, Jose Trevino, Edward Agyare

**Affiliations:** 1 College of Pharmacy and Pharmaceutical Sciences, Florida A & M University, 1415 South Martin Luther King Blvd, Tallahassee, FL 32307, USA.; 2 The National High Magnetic Field Laboratory, Florida State University, Tallahassee, FL, USA.; 3 Department of Chemical & Biomedical Engineering, FAMU-FSU College of Engineering, Florida State University, Tallahassee, FL, USA.; 4 Keck School of Medicine University of Southern California, Los Angeles, USA.; 5 Department of Surgery, University of Florida Medical Center, Gainesville, FL, USA.; 6 Present Address: Food and Drug Administration, Silver Spring, MD, USA.

**Keywords:** Nanoparticle, Gadolinium, Magnevist®, Pancreatic cancer, Patient-derived xenograft, Magnetic resonance imaging

## Abstract

**Background:**

The aim of this study was to compare contrast enhancement of Magnevist® (gadopentate dimeglumine (Mag)) to that of PEGylated Magnevist®-loaded liposomal nanoparticles (Mag-Lnps) in pancreatic cancer patient-derived xenograft (PDX) mouse model via magnetic resonance imaging (MRI).

**Methods:**

Mag-Lnps formulated by thin-film hydration and extrusion was characterized for the particle size and zeta potential. A 21.1 T vertical magnet was used for all MRI. The magnet was equipped with a Bruker Advance console and ParaVision 6.1 acquisitions software. Mag-Lnps phantoms were prepared and imaged with a 10-mm birdcage coil. For in vivo imaging, animals were sedated and injected with a single dose (4 mg/kg) of Mag or Mag-Lnps with Mag equivalent dose. Using a 33-mm inner diameter birdcage coil, *T*_1_ maps were acquired, and signal to noise ratio (SNR) measured for 2 h.

**Results:**

Mag-Lnps phantoms showed a remarkable augmentation in contrast with Mag increment. However, in in vivo imaging, no significant difference in contrast was observed between Mag and MRI. While Mag-Lnps was observed to have fairly high tumor/muscle (T/M) ratio in the first 30 min, free Mag exhibited higher T/M ratio over the time-period between 30 and 120 min. Overall, there was no statistically significant difference between Mag and Mag-Lnp in rating MR image quality. Low payload of Mag entrapment by Lnps and restricted access of water (protons) to Mag-Lnps may have affected the performance of Mag-Lnps as an effective contrast agent.

**Conclusion:**

This study showed no significance difference in MRI contrast between Mag and Mag-Lnp pancreatic cancer PDX mouse models.

## Background

There has been an effort toward the use of contrast agents packaged as nanoparticulate systems to enhance contrast in imaging of solid tumors via MRI (Pan et al. 2010). To ascertain if nanoparticle-loaded contrast agents would exhibit higher contrast than free contrast agent in an unperturbed solid tumor environment, a comparative study was conducted on MR imaging of tumors using free Mag and Mag-Lnp. Although Mag, which was approved by Food and Drug Administration (FDA), has been widely used in clinical practices as MRI contrast agent (Raatschen et al. 2006), Mag has exhibited some limitations such as short blood circulation time, relatively low relaxivity and potential toxicity, for example, nephrogenic systemic fibrosis. On the other hand, nano-contrast agents present distinct advantages over traditional MRI contrast agents as they have been reported to displayed superiority in in vitro imaging, prolong systemic circulation and provide optimum window for imaging (Ghaghada et al. 2008).

A circumspective review of traditional contrast agents which are usually gadolinium-based contrast agents (GBCA) has revealed that these agents are nonspecific and undergo rapid extravasation into extracellular compartment with rapid elimination from the body (Aime and Caravan 2009; Ghaghada et al. 2009). Another pertinent challenge which precludes traditional imaging in MRI with GBCA is the difficulty in detecting tumors with diameter 1 cm or less due to low resolution (Fisher et al. 2001). As a result, obscured tumors go undetected via MRI (Eloy et al. 2014). To improve on contrast enhancement of such tumors, functionalized and biocompatible nano-delivery system with high payload of gadolinium (Gd) can deliver and retain high level of gadolinium (Gd) through enhanced permeation and retention (EPR) effect that exists preferentially in tumors (Dewi et al. 2013).

More importantly, tumors that exhibit EPR effect at very early stages (tumor size of 2–3 mm) of growth due to neoangiogenesis tend to accumulate more nanoparticles (Eloy et al. 2014). Further, liposomal nanoparticle, which has the ability to navigate, retain and release its content into tumor site, has been investigated as a prospective nano-contrast agent for high contrast (Hossann et al. 2013; Na et al. 2011). Report shows studies, that have employed contrast agents in thermosensitive nanoparticles as a way to monitor its content or drug release, have also exhibited high MR contrast in *T*_1_-weighted images using the same nanoparticles (Lorenzato et al. 2016). In another study, liposomal nanoparticles demonstrated high residence time and high relaxivities for Gd chelates compared with unencapsulated Gd chelates in vivo (Tian et al. 2017). It also enhances T_1_ relaxivity and improves signal to noise ratio (SNR) (Ghaghada et al. 2009).

Numerous reports show data where paramagnetic metal oxides-loaded nanoparticles exhibited low toxicity and improved contrast in tumors compared with free paramagnetic metal oxides. The examples include (i) PEGylated gadolinium oxide (Gd_2_O_3_) nanoparticles versus Mag (Ahren et al. 2010; Dai et al. 2018) and (ii) gadolinium (Gd ions)-loaded chitosan nanoparticles versus Mag (Zhang et al. 2013). Despite the extensive studies on contrast enhancement using free contrast and contrast agent-loaded nanoparticles, there was no datum or publish paper that compared contrast enhancement in tumor using Mag and Mag-Lnp in pancreatic cancer PDX mouse model through MRI.

While PCa remains one of the most deadly cancers in USA with 95% mortality on the average within the first 6 months after diagnosis (Farrow et al. 2008), the quest for early detection, modulation of dense desmoplastic stroma to improve drug penetration and eventually enhance therapeutic efficacy remains a herculean task to both researcher scientists and clinicians (Farrow et al. 2008; Mei et al. 2016).

As intimated, tumor size of 1 cm or less is difficult to detect and targeting functionalized contrast-loaded nanoparticle to relevant biomarkers such as carbohydrate antigen-19–9 (CA19–9) has not been successful due to low expression of markers, nonspecificity or non-selectivity (Eloy et al. 2014; Hanada et al. 2015).

Nanoparticles of Gd(III)-based MRI contrast agents have been formulated and investigated their ability to enhance MRI contrast in tumors derived from commercially available cells. However, these are highly passaged cancer cell lines with limited translational value (Oyewumi and Mumper 2003; Pham et al. 2016; Stefancikova et al. 2016; Sun et al. 2016; Swanson et al. 2008). In our literature search, we did not come across any nano-contrast agents studies that focused on pancreatic cancer PDX model. Such studies are important because PDX models preserved, in part, the intratumoral heterogeneity and complex biological barriers known to exist in PCa (Pham et al. 2016).

In this current work, a proof-of-concept study was conducted. The objective was to evaluate contrast enhancement of Mag and Pegylated Mag-Lnps in pancreatic cancer PDX mouse models via MRI while mice remained alive. MR imaging at 21.1 T was employed because of its ability to produce high signal-to-noise ratio with improved resolution at a faster rate compared to lower magnetic field strength that would require long period of time with a lower signal-to-noise ratio. Mag was compared with Mag-Lnps in terms of changes in *T*_1_ relaxivity and SNR (tumor and surrounding muscles). For emphasis, we adopted pancreatic cancer PDX model as it represents human tumor with a more reliable predictive value and a well-preserved morphological characteristic of patient tumor specimen (Delitto et al. 2015; Pham et al. 2016).

## Results

### Characterization of Mag-Lnps

It is well documented that certain physiological processes such as accumulation, tissue diffusion, tissue extravasation and kidney excretion largely depend on size of particles and sizes (≤ 140 nm) are able to enter and exit fenestrated capillaries in the tumor microenvironment (Bertrand and Leroux 2012; Blasi et al. 2007). The mean particle size of free Mag determined by dynamic light scattering method was found to be in the range of 16.5–17.3 nm while Mag-Lnps mean size ranged from 167.3 to 173.5 nm (Table 1). While the net surface charge (zeta potential) of the Mag-Lnps was moderately positive 2.28 ± 0.19, net surface charge of Mag was found to be − 1.86 ± 0.05 (Table 1). For polydispersity index (P.I), both Mag (0.61 ± 0.01) and Mag-Lnps (0.17 ± 0.03) exhibited a narrow particle width distribution with moderately uniform particle size population.

### TEM analysis for Mag-Lnps

**Fig. 1:**
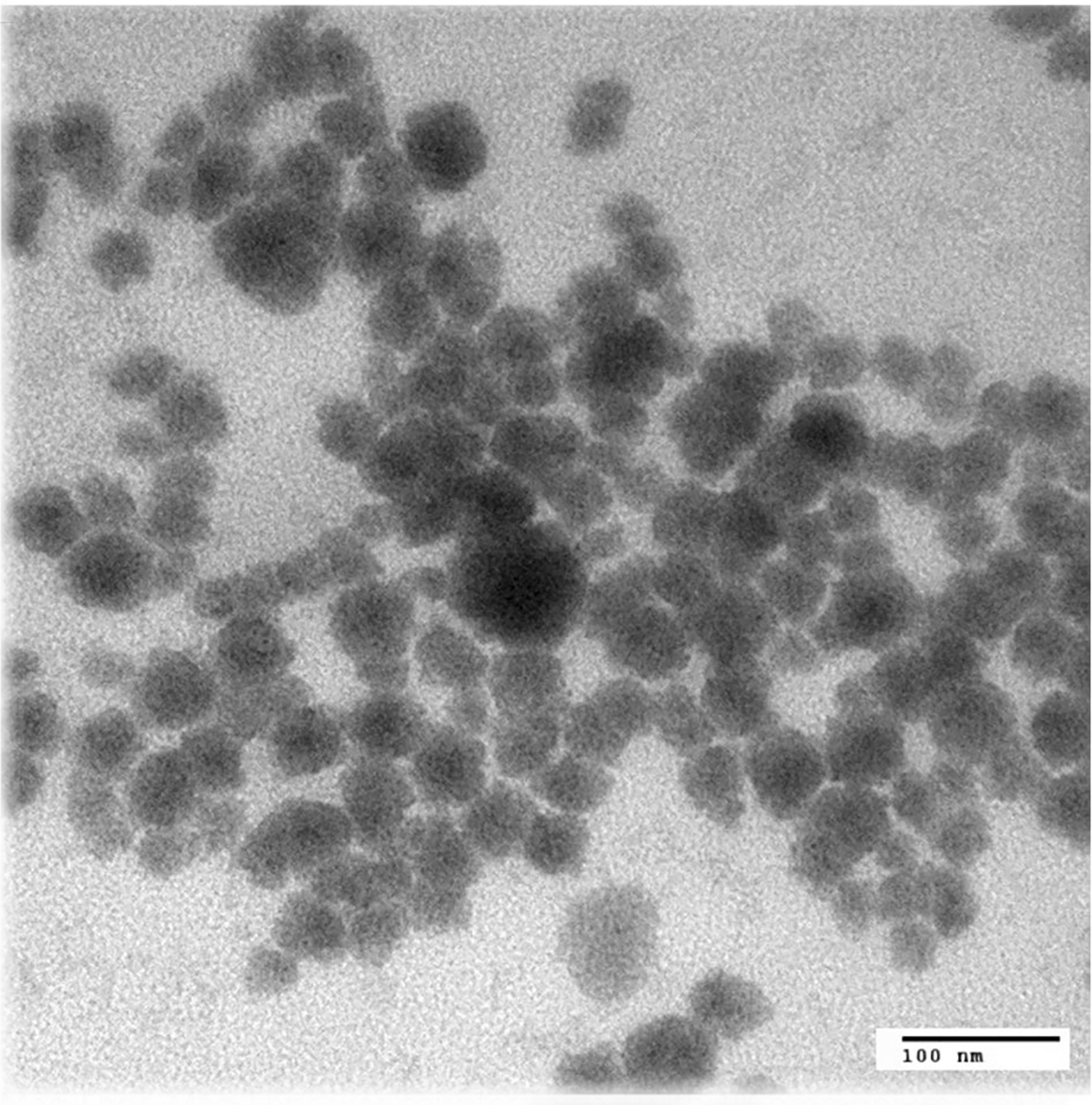
TEM analysis of Mag-Lnps. Mag-Lnps was counterstained with 1% ammonium molybdate

As expected, TEM images of Mag-Lnps exhibited somehow spherical structures as shown in Fig. 1 with sizes slightly smaller than the average particle hydrodynamic diameter measured by principle of dynamic light scattering (DLS) in Table 1. The DLS measurements reveal the true state of particles in media or solution. In the medium, a thin dipole layer of the solvent adheres to the particle’s surface which provides additional size to the particle core leading to a higher hydrodynamic diameter. While in an estimated size by TEM, this hydration layer is not present; hence, the TEM provides information only about the core of the primary particle size.

### MRI of Mag-Lnps phantoms

**Fig. 2:**
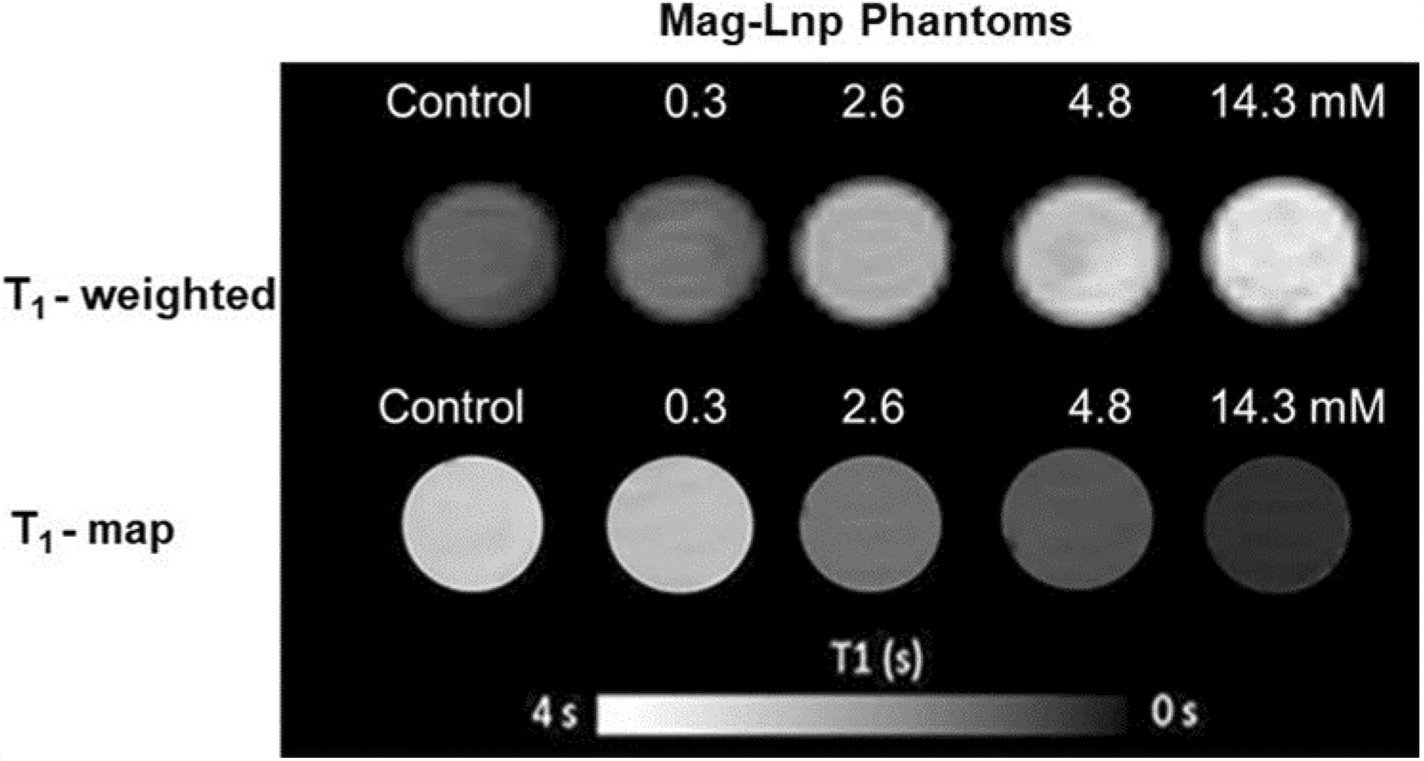
Compared contrast agents of phantoms. The *T*_1_-weighted images of Mag-Lnps phantoms displayed positive relation between Mag-Lnp phantom and contrast enhancement

**Fig. 3:**
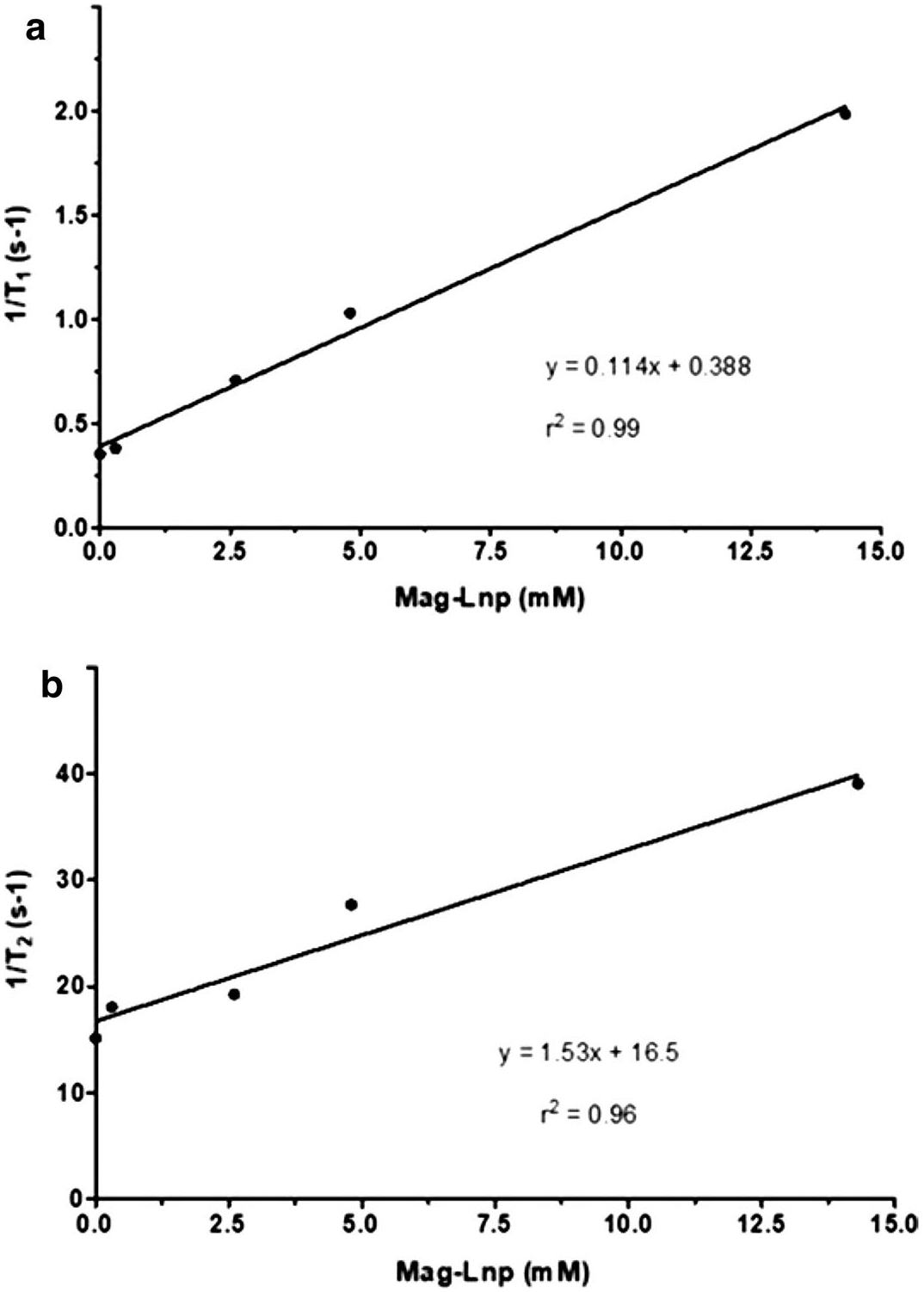
1/*T*_1_ measured with phantoms of various concentrations of Mag-Lnp at 37 °C (**a**), 1/*T*_2_ measured with phantoms of various concentrations of Mag-Lnp at 37 °C (**b**). Relaxivity was calculated as the slope of 1/*T* vs Mag-Lnp concentration

In the relaxometry study, we measured *T*_1_ and *T*_2_ values of phantoms as a function of Mag-Lnps concentration (Figs. 2 and 3). The main purpose of the agarose was to mimic a tissue and the contrast monitored with respect to increment in Mag-Lnps concentration. As observed in Fig. 2, the contrast of *T*_1_ image enhances with increase in Mag-Lnps concentration. A noticeable contrast was first observed at a concentration of 2.6 mM with the highest contrast exhibited at 14.3 mM. Contrast was virtually absent in 0.3 mM Mag-Lnps phantom and comparable to that of the control image (Fig. 2). Graphs of the longitudinal (1/*T*_1_) or transverse (1/*T*_2_) water relaxation rates versus the concentrations of the contrast agent Mag-Lnp are shown in Fig. 3a, b. The relaxivities, *R*_1_ and *R*_2_, were found to be 0.11 and 1.53 mM^−1^s^−1^, respectively, as shown in Table 2. These relaxivities were relatively low compared to that of free Mag where *R*_1_ and *R*_2_ was between 3.0–3.4 mM^−1^s^−1^ and 3.8–4.2 mM^−1^s^−1^ respectively at 4.7 T (Rohrer et al. 2005; Zhang et al. 2003). There was strong relation between 1/*T*_1_ and Mag-Lnp concentration with r ^2^ value of 0.99, while relation between 1/*T*_2_ and Mag-Lnp concentration followed a similar trend with *r*^2^ value of 0.96 (Fig. 3a, b) (Agyare et al. 2014).

### In vivo imaging of pancreatic PDX via MR

**Fig. 4:**
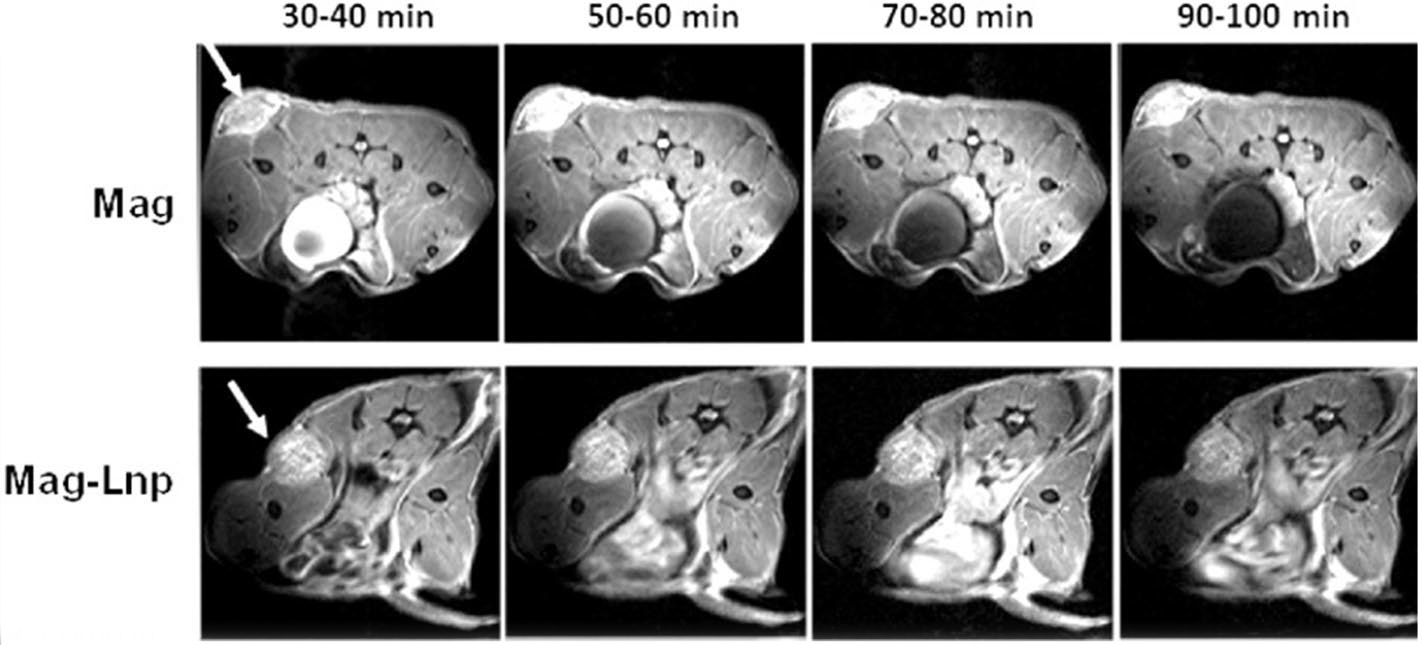
Comparison of contrast enhancement (*T*_1_) of Mag and Mag-Lnp pancreatic PDX tumors as a function of time after single bolus intravenous administration of Mag and Mag-Lnp

As shown in Fig. 4, MR scans (*T*_1_ images) of Mag and Mag-Lnps tumor images were compared after intravenous injection of Mag and Mag-Lnps with dose equivalent of Mag. By careful examination of obtained tumor images, Mag-Lnps injected tumors exhibited moderate increase in contrast enhancement. But the contrast was not statistically significant when compared with free Mag-treated mice (Fig. 4).

**Fig. 5:**
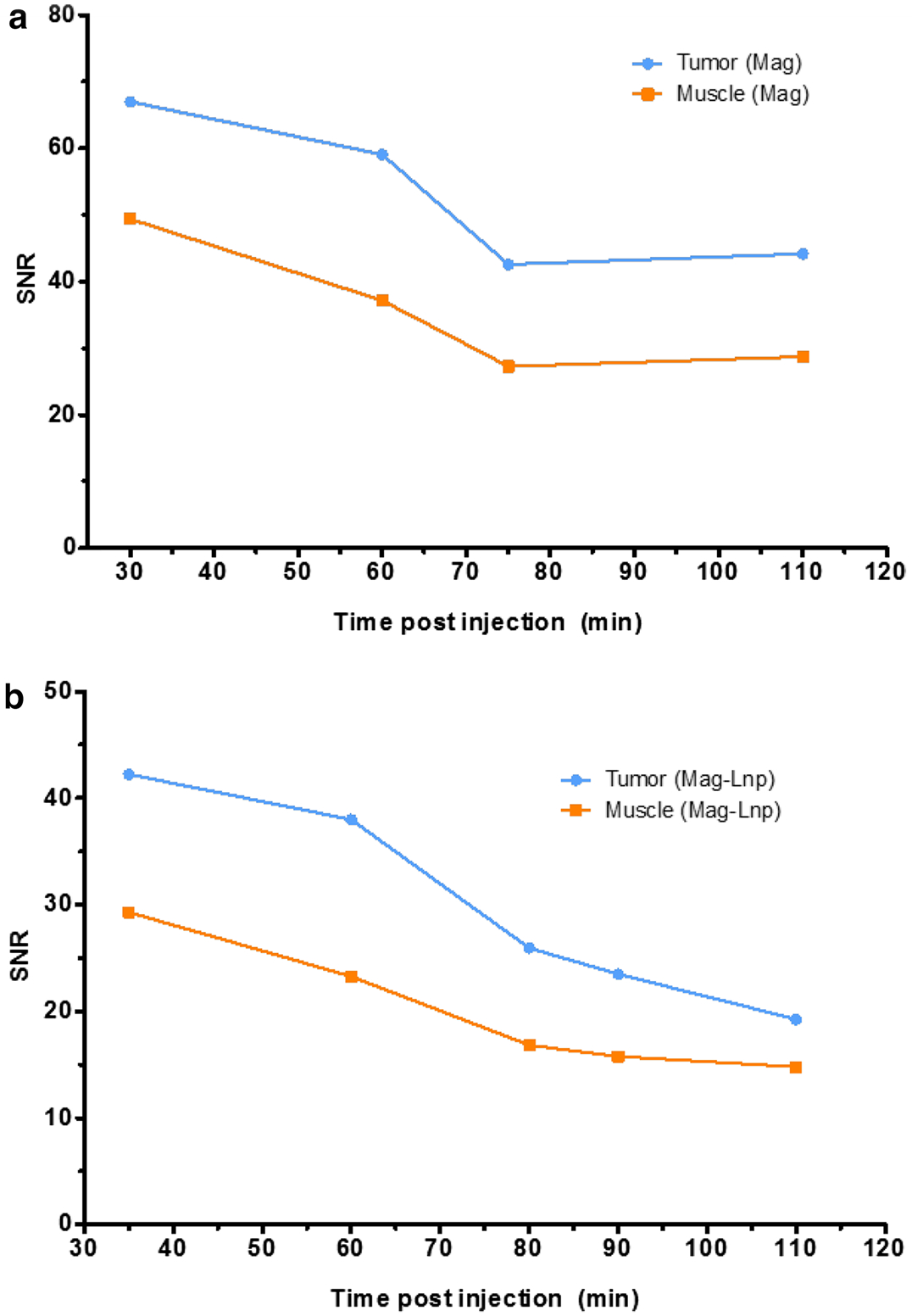
Signal-to-noise ratio (SNR) in tumor and surrounding tumor muscle. Relation between SNR and Mag after single bolus intravenous administration Mag (**a**), relation between SNR and Mag after single bolus intravenous administration Mag-Lnp (**b**)

Also, dynamic changes in contrast were monitored in tumor with respect to the surrounding muscle after Mag and Mag-Lnps were administered. In Fig. 5, a general decline in SNR was observed between Mag and Mag-Lnps. However, Mag showed increase in SNR between the 70 and 80 min time points. This suggests that Mag might have diffused back into tumor hence the slight increase in SNR.

Further, ratio of signal of tumor (T) to muscle (M), (T/M), was determined and the data did not reveal any remarkable difference in T/M values of Mag and Mag-Lnps groups (Table 3).

**Fig. 6:**
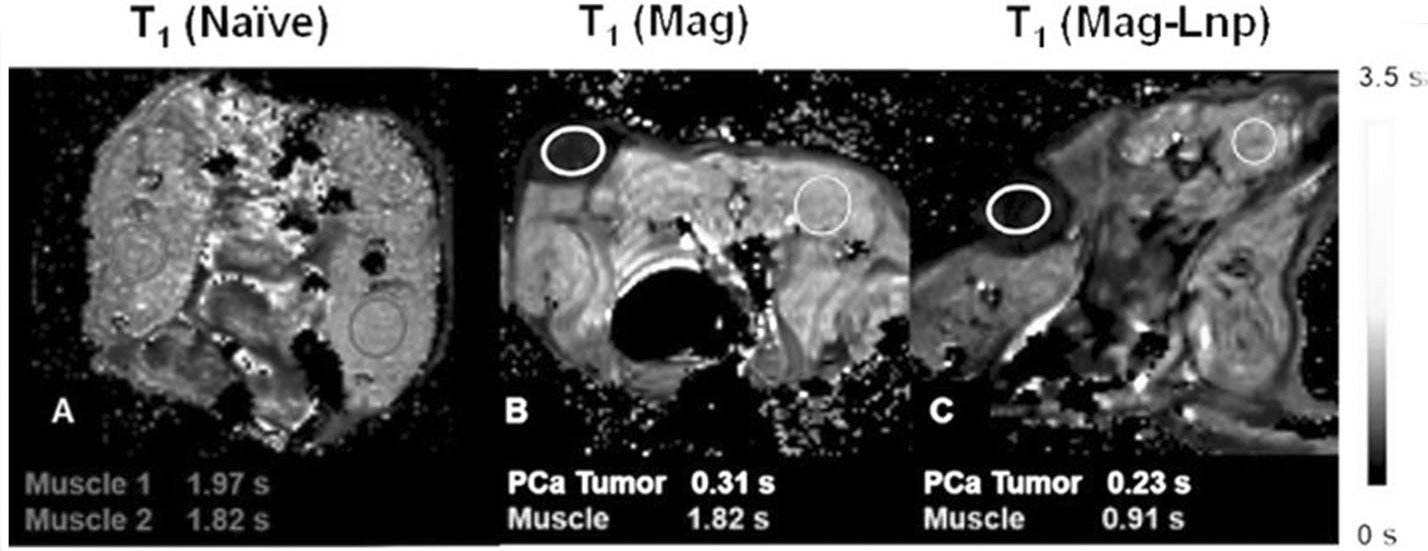
In vivo *T*_1_-maps of naïve, Mag and Mag-Lnp PCa PDX. The MR images were captured 30 min after single bolus intravenous administration Mag and Mag-Lnp. Relaxation times were extracted with ROIs in the tumor and surrounding tissue (muscles)

To assess the possibility of contrast difference between Mag and Mag-Lnps in the tumors, *T*1 relaxation was mapped and regions of interest (ROIs) drawn within the tumor. These ROIs included the tumor tissue and its surrounding muscle tissues as shown in Fig. 6. The *T*_1_ relaxation time of the tumor as circled in yellow for Mag was not significantly different from that of Mag-Lnps. Notably, *T*_1_ mapping relaxation time in the muscle region (circled in red) was increased by approximately twofold in animal that received Mag (1.82 s) injection compared with Mag-Lnps (0.91 s) signifying reasonable uptake of Mag as compared with Mag-Lnps.

## Discussion

Detection of pancreatic tumor at an early stage is a substantial challenge especially when the tumor is less than 10 mm and it sheds no clinically relevant biomarker in the early stages. In fact, early detection of tumor has shown favorable prognosis as surgical resection is highly probable. In view of this, imaging modalities such as MRI has played a remarkable role in early-stage detection and characterization (Hanada et al. 2015).

Gadolinium (Gd) chelates, a low molecular weight marker, has found immense application in tissue contrast and diagnostic efficiency in magnetic resonance scans by prolonging the relaxation rate (1/*T*_1_) of fluid protons (Fretellier et al. 2015). But one key issue with MR imaging is the rapid clearance and non-specific distribution of Gd and this has necessitated the development of smart delivery systems to ferry contrast agents into specific tissue or disease of interest (Liu and Zhang 2012). This study sought to compare the MRI contrast enhancement of Mag to that of Mag-Lnps in PCa PDX mouse model monitored by MRI.

First, Mag-Lnp particle hydrodynamic diameter was determined to have a desirable size of 170.4 ± 3.12 nm which was an idea size to pass through endothelial gaps found within tumor vasculature which have been reported to be in the typical range of 100–500 nm (Azzi et al. 2013; Hashizume et al. 2000). Nonetheless, TEM analysis revealed particle sizes less or equal to 100 nm. A literature search reveals that principle of measurements involved in TEM and DLS analyses is greatly different and can influence the variations observed in particle sizing measurements (Souza et al. 2016). In principle, DLS measurement involves light scattering by the nanoparticles which seems to suggest that larger particles are more likely to scatter more light. This simply means that DLS preferentially measures larger particles in a hydrodynamic fashion. While TEM measurements actually measure the “true” size and reveal the morphology of the nanoparticles (Ito et al. 2004). As reported by others, particle size of not more than 200 nm has been found to effectively extravasate passively into tumor interstitium (Sun et al. 2014). Although, the particle size of Mag-Lnp is of optimal size for passive targeting, the zeta potential (2.28 ± 0.19 mV) negatively impacts the circulation of the Mag-Lnps as the positively charged nanoparticles are easily recognized by (MPS) and could lead to rapid clearance from blood.

Measurement of relaxation rate as a function of Mag concentration in Mag-Lnps phantoms showed an increased in MR signal or contrast with increase in concentration, signifying a potential use as an MRI contrast agent. But the low T_1_ relaxivity value of 0.11 s^−1^ mM^−1^ displayed by Mag-Lnp may raise questions about Mag-Lnp’s full potential as an MRI contrast agent. There are reasons that can be attributed to the low T_1_ relaxivity such as rapid elimination of Mag-Lnp as intimated, rapid diffusion of Mag-Lnp from tumor vasculature back to systemic circulation, and low payload of Mag in Lnps. In the mechanism of MRI contrast enhancement, interaction between protons contributed by water or in the microenvironment and contrast agent is of prime importance, but our formulation technique entrapped more Mag into the core of the liposomal nanoparticle which restricted the free shuttling of protons (H +) across the lipid layer of the liposomal nanoparticles to interact with Mag. Based on this, we may infer that lack of free interaction between proton and Mag might have resulted in a low or moderate MRI signal or T1 relaxation (Lim et al. 2014).

We acknowledged the fact that various nanoparticles-based MRI contrast agents such as Gd(III) and Mag have been developed and investigated their ability to enhance MRI contrast using largely commercially available, highly passaged PCa cell lines which are of limited translational value but have shown improved contrast (Oyewumi and Mumper 2003; Stefancikova et al. 2016; Sun et al. 2016; Swanson et al. 2008). None of these studies used PDX model which preserve, in part, the intratumoral heterogeneity known to exist in PCa (Pham et al. 2016). In addition, we believe that the tumor architecture of pancreatic cancer confers a barrier that is barely surmounted to large molecules. Essentially, pancreatic cancer tumor is characterized by a dense desmoplastic stroma which is enclosed by a fibrotic connective tissue significantly diminishes the penetration of large macromolecules like nanoparticles. The overarching impact is the increased interstitial fluid pressure in these solid tumors and subsequently contributed to the observed trend in animals exposed to Mag-Lnp. As observed, contrast enhancement was not increased appreciably in the primary patient tumor in contrast to previous studies that ultimately investigated tumors derived from commercially available tumors. For emphasis, we did not come across any reports that compared free Gd(III)- or Mag-based contrast agent to its corresponding loaded nanoparticles in PDX mouse models in real-time imaging.

## Conclusion

This study highlighted Mag-Lnps modest increase in MRI contrast enhancement in pancreatic PDX mouse imaging compared with that of free Mag. We hold the belief that the positively charged surface of Mag-Lnps and PCa stromal barriers may have reduced deeper penetration and intratumoral distribution of Mag-Lnps.

In our quest to develop a novel Mag-loaded nanoparticle that would exhibit high contrast enhancement, we plan to develop four different PEGylated Mag-containing nanoparticles, namely (i) Mag-loaded nanoparticle, (ii) Mag surface-modified nanoparticle, (iii) Mag-loaded nanoparticle with surface-modified Mag, and (iv) Mag-loaded nanoparticle with surface-modified ligand, and investigate their potential to significantly enhance image contrast. To disrupt the stromal barriers, we plan to pretreat pancreatic PDX mouse models with hyaluronidase prior to Mag-loaded nanoparticles’ administration. This will propitiously improve the delivery and contrast enhancement of Mag-loaded nanoparticles in the tumor.

## Materials and methods

Dipalmitoyl phosphatidylcholine (DPPC), 1-myristoyl-2-palmitoyl-sn-glycero-3-phosphocholine (MPPC) and 1,2-distearoyl-sn-glycero-3-phosphoethanolamine-*N*-[amino(polyethylene glycol) 2000] (DSPE-PEG_2000_) were bought from Avanti Polar Lipids (Alabaster, AL). Mag (gadopentate dimeglumine) was bought from Bayer HealthCare Pharmaceuticals Inc. (Wayne, NJ). All solvents used were of analytical grade.

### Preparation and characterization of Mag-Lnps

The fabrication of Mag-Lnps was adopted from previous methods and other publications (Affram et al. 2015; Ghaghada et al. 2008, 2009). In brief, DPPC, MPPC and DSPE-PEG_2000_ in the ratio 90:10:4 respectively were weighed to yield a total weight of 100 mg. Subsequently, lipids were dissolved in chloroform followed by the removal of chloroform by drying the lipid mixture solution in a stream of dry nitrogen gas under a fume hood.

Residual chloroform in the thin lipid mixture was further removed by incubating the mixture in a vacuum chamber for 2 h. The thin film was then hydrated by vortexing it with 2 mL (an aliquot fashion) of PBS solution containing 37.5 mM Mag to yield a suspension of multilaminar vesicles (MLV). The lipid suspension was then extruded (11 times) through a 200-nm polycarbonate membrane placed between two filter supports.

This process was repeated using 100-nm polycarbonate membrane. The temperature of the heating block was kept below 80 °C while the liquid suspension was kept between 55 and 60 °C (above the phase transition of the liquid) during hydration and extrusion.

The formed liposomes solution was diluted with distilled water according to the manufacturer’s instructions and the particle size and zeta potential determined on the NICOMP^™^ Particle Sizing Systems (Santa Barbara, California, USA).

### Transmission electron microscopy (TEM) measurement of Mag-Lnps

As part the physicochemical characterization of Mag-Lnps, the structural morphology of Mag-Lnps was determined by TEM (JEOL) at 120 kV. A drop of the Mag-Lnps prepared as described above, serially diluted and placed on a carbon-coated copper grid and negatively stained with 1% ammonium molybdate. The grid was dried and viewed by JEM-ARM200cF TEM.

### MRI measurements

MRI scans were carried out on the ultra-wide bore 21.1 T (900 MHz) vertical magnet built at the National High Magnetic Field Laboratory (NHMFL) (Fu et al. 2005). The magnet is equipped with a Bruker Avance III console and data acquisition was performed with ParaVision 6.0.1 acquisition and processing (BioSpinCorp, Billerca, MA) together with a 64-mm inner diameter high-performance gradient (Resonance Research Inc, MA) capable of producing 0.6 T/m peak gradient strength.

### Mag-Lnp phantom imaging via MRI

Mag-Lnps phantoms were prepared as described by others (Affram et al. 2017; Agyare et al. 2014). In brief, Mag-Lnps solution was diluted by thoroughly mixing it with distilled water in a ratio of 1:1, 1:5, 1:10 and 1:100. The various Mag-Lnps diluted solutions were further mixed with 1% agarose solution in the ratio 1:1 to yield concentrations 14.3 mM (1:1), 4.8 mM (1:5), 2.6 mM (1:10) and 0.3 mM (1:100), warmed slightly and carefully loaded into microcapillary tube. After solidification, the ends of each tube were sealed with wax.

For control, equal volume of distilled water and 1% agarose was prepared, loaded into microcapillary tube and allowed to solidify. All test samples and control were stored at 4 °C overnight prior to MR imaging (Agyare et al. 2014). Using a 10-mm birdcage radio frequency (RF) coil, Mag-Lnps phantoms together with control were loaded together and measurements were made to determine 1/*T*_1_ (*R*_1_) and as 1/*T*_2_ (*R*_2_) relaxation rates. Data acquisition was achieved with a 100 × 100 matrix in a plane resolution of 100 × 100 μm with a slice thickness of 1 mm. A spin echo (SE) sequencing using nine (9) incrementing repetition times (TR) between 26 and 15,000 ms and 16 incrementing echo time (TE) between 10 and 160 ms was performed to obtain R_1_ and R_2_, respectively. Signal intensity was used to determine R_2_ relaxation rates by mapping regions of interest (ROIs) from the sample scan against TE using a single exponential decay function.

### Animals

Female PDX mice (NOD/Scid-IL2rg) models with tumor planted in the left flank were used and all procedures with mice were in strict accordance with the National Institutes of Health Guide for the Care and Use of Laboratory Animals and approved by the Florida A & M University Animal Care and Use Committee. Mice were put up in a virus-free, indoor, light and temperature controlled barrier environment with unlimited access to water and food.

### In vivo imaging of pancreatic PDX via MR

The mice bearing pancreatic PDX were injected with single dose (4 mg/kg) of Mag or Mag-Lnps equivalent dose of Mag through intraperitoneal (IP) route after the average tumor size has reached 12 mm. Prior to imaging, mice were sedated with isoflurane in an enclosed chamber. Following sedation, tumor-bearing mouse was secured with a home-built 33-mm inner diameter RF birdcage coil with tumor positioned at center of the coil. Data acquisition (T_1_ maps) was acquired with 250 × 210 mm in-plane resolution and 0.75 mm slice thickness (Fig. 6). TR was incremented 6 times between 170 and 4000 ms and TE was 6 ms. SNR was measured over time with a turbo spin echo using TE/TR = 5/1500 ms and 90 × 90 mm in plane resolution and a 1-mm slice. Both acquisitions took 7 min.

### Statistical analysis

Data characterizing Mag and Mag-Lnps was presented as mean ± standard deviation and statistical difference between Mag and Mag-Lnps was determined using Student’s *t* test and considered significant at *p* < 0.05. With the exception of in vivo MRI and MRI phantoms, all other experiments were performed at least in triplicate and analyzed using GraphPad Prism software (GraphPad Software, Inc., La Jolla, CA, USA).

## Figures and Tables

**Table 1:** Mean particle size, zeta potential and polydispersity index (P.I) of Mag and Mag-Lnps

Formulation	Mean particle size (nm)	Mean zeta potential (mV)	Polydispersity index (P.I)
Mag	16.9 ± 0.4	− 1.86 ± 0.05	0.61 ± 0.01
Mag-Lnps	170.4 ± 3.1	2.28 ± 0.19	0.17 ± 0.03

Data represent mean ± SD, *n* = 3 (*Mag* Magnevist, *Mag*-*Lnps* magnevist-loaded liposomal nanoparticles)

**Table 2:** Gadolinium (Gd) concentration (mM) and respective *T*1 and *T*2 relaxation times

Mag-Lnps (mM, Gd)	*T*_1_ (ms)	*T*_2_ (ms)
14.3	503.1	25.6
4.8	970.9	36.1
2.6	1414.4	51.8
0.3	2638.5	55.3
Control (agarose)	2849.0	66.1
Relaxivity (s^−1^mM^−1^)	0.11	1.53

**Table 3:** Measurement of the relative tumor-muscle ratio (SNR_tumor_/SNR_muscle_) with respect to time

Mag		Mag-Lnp	
Time (min)	T/M	Time (min)	T/M
30–40	1.35	30–40	1.48
50–60	1.58	50–60	1.63
70–80	1.56	70–80	1.53
90–100	1.54	90–100	1.49
110–120	1.77	110–120	1.30

*T* tumor, *M* muscle, T/M ratio
